# Comparison of the perfusion index as an index of noxious stimulation in monitored anesthesia care of propofol/remifentanil and propofol/dexmedetomidine: a prospective, randomized, case-control, observational study

**DOI:** 10.1186/s12871-023-02116-x

**Published:** 2023-05-26

**Authors:** Doyeon Kim, Changjin Lee, HanWool Bae, Jeayoun Kim, Eun Jung Oh, Ji Seon Jeong

**Affiliations:** 1 Department of Anesthesiology and Pain Medicine, CHA Bundang Medical Center, CHA University School of Medicine, Seongnam, Korea; 2 Department of Anesthesiology and Pain Medicine, Samsung Medical Center, Sungkyunkwan University School of Medicine, Seoul, Korea; 3 Department of Anesthesiology and Pain Medicine, Chung-Ang University Gwangmyeong Hospital, Chung-Ang University Scholl of Medicine, Gwangmyeong, Korea; 4 Department of Anesthesiology and Pain Medicine, Samsung Medical Center, Sungkyunkwan University School of Medicine, 81 Irwon-ro, 06351 Gangnam, Seoul, Korea

**Keywords:** Ambulatory surgery, Analgesia, Dexmedetomidine, Monitored anesthesia care, Perfusion index, Remifentanil

## Abstract

**Background:**

Dexmedetomidine, one of the sedatives, has an analgesic effect. We aimed to investigate postoperative analgesia with dexmedetomidine as adjuvants for procedural sedation using perfusion index (PI).

**Methods:**

In this prospective, randomized, case-control, observational study, 72 adult patients, 19–70 years, who were scheduled for chemoport insertion under monitored anesthesia care were performed. According to the group assignment, remifentanil or dexmedetomidine was simultaneously infused with propofol. The primary outcome was PI 30 min after admission to the post anesthesia care unit (PACU). And, pain severity using numerical rating scale (NRS) score and the relationship between NRS score and PI were investigated.

**Results:**

During PACU staying, PI values were significantly different between the two groups PI values at 30 min after admission to the PACU were 1.3 (0.9–2.0) in the remifentanil group and 4.5 (2.9–6.8) in the dexmedetomidine group (median difference, 3; 95% CI, 2.1 to 4.2; *P* < 0.001). The NRS scores at 30 min after admission to the PACU were significantly lower in the dexmedetomidine group (*P* = 0.002). However, there was a weak positive correlation between NRS score and PI in the PACU (correlation coefficient, 0.188; *P* = 0.01).

**Conclusion:**

We could not find a significant correlation between PI and NRS score for postoperative pain control. Using PI as a single indicator of pain is insufficient.

**Trial registration:**

Clinical Trial Registry of Korea, https://cris.nih.go.kr: KCT0003501, the date of registration: 13/02/2019.

## Introduction

Monitored anesthesia care is an anesthetic method applied in relatively simple surgeries, especially in ambulatory surgeries that do not require hospitalization. For adequate sedation, anesthesiologists choose various combinations of sedatives and analgesics. Remifentanil, a µ-opioid, is widely used to induce anesthesia, sedation, and analgesia during anesthesia maintenance. Because of its ultra-short half-life, remifentanil does not exhibit a residual opioid effect after discontinuation of administration [1]. Dexedetomidine is a highly selective α-2 agonist that is popular in clinical practice along with remifentanil. It not only has a sedative effect, but also has analgesic sparing effect [2]. We also have demonstrated that dexmedetomidine was effective for postoperative pain management in a previous study [3]. Although relatively simple surgeries are performed in the form of ambulatory surgery, it has been reported that 30–40% of patients undergoing ambulatory surgery experience moderate to severe pain in the first 24–48 h after surgery [4]. Therefore, adequate analgesia after ambulatory surgery is also important.

The perfusion index (PI) is a device that measures the ratio between pulsatile and non-pulsatile blood flow [5]. Moreover, when the sympathetic nervous system is stimulated due to pain, the PI value decreases due to an increase in vasomotor tone or constriction of peripheral blood vessels [6]. Previous study demonstrated that postoperative pain reduction was associated with increased PI in the post anesthesia care unit [7]. These indicate that PI may serve as an indicator for pain assessment. We hypothesized that the combined use of dexmedetomidine and propofol during monitored anesthesia care would result in adequate postoperative analgesia. In addition, it was assumed that this analgesic effect would be associated with a high PI. Accordingly, we investigated the analgesic effect of remifentanil and dexmedetomidine as an adjuvant for procedural sedation and to evaluate between PI and pain severity.

## Materials and methods

### Ethics approval and consent to participate

This prospective, randomized, case-control, observational study was approved by the Samsung Medical Center Institutional Review Board, Samsung Medical Center, Seoul, Republic of Korea (Chairperson: Prof. Young Keun On) on 6th, December 2018 (no. SMC 2018-11-111). Prior to the recruitment of the first participants, we registered them in the Clinical Trial Registry of Korea (https://cris.nih.go.kr; registration no. KCT0003501, principal investigator: Ji Seon Jeong; date of first registration:13/02/2019). Written informed consent was obtained from all participants before enrolment in the study. All methods were performed in accordance with the Declaration of Helsinki and its revisions.

### Patients and protocols

Adult patients aged 19–70 years with American Society of Anesthesiologists physical status I and II scheduled for elective chemoport insertion under monitored anesthesia care were assessed for eligibility and were included between February 2019 and December 2019. Exclusion criteria were allergy to certain anesthetic agents; body mass index ≥ 30 kg/m^2^; diabetes mellitus; cardiovascular disease (e.g., myocardial infarction, heart failure, or arrhythmia including bradycardia ≤ 50 bpm); use of α/β blocking agents; pre-existing chronic pain; use of analgesics; peripheral occlusive artery disease; severe liver or kidney disease; or pregnancy.

### Randomization and group allocation

One statistician who was not involved in this trial generated a random allocation sequence using permuted-block randomization with a block size of 4. Randomization and group allocation were performed in a 1:1 ratio using the sealed opaque envelope technique. The study drugs were prepared in a 20 ml syringe depending on the group assigned:

#### Remifentanil group

remifentanil 1 mg (Remiva Inj., Remifentanil Hydrochloride, Hana Pharm Co., Ltd.) + 0.9% saline 20 ml.

#### Dexmedetomidine group

dexmedetomidine 80 μg (premixed solution of 4 mcg/ml) (Precedex Inj. Dexmedetomidine Hydrochloride, Pfizer Ltd.)

### Protocol

Premedication was not permitted for all participants. The prepared study drug was delivered to a clinical anesthesiologist (unrelated to this study) just before the start of anesthesia induction. On arrival in the operating room, standard monitors, including electrocardiograms, pulse oximetry, and noninvasive blood pressure (NIBP), were applied. A PI sensor (Radical-7®; Masimo Corporation, Irvine, CA, USA) was attached to the index finger and the finger was covered with a black opaque pouch to minimize light interference. The body temperature of all patients in our institute was maintained above 36.5 °C in the operating room using circulating-water blanket. The temperature of the operating room and PACU was maintained as 21 and 23 °C to prevent the influence of environmental temperature. In addition, to prevent the influence on blood flow, the NIBP was measured on the opposite side of the attachment site of the PI sensor.

The degree of sedation was evaluated using the modified observer’s assessment of alertness/sedation (OAA/S) scale. To maintain OAA/S scale of 3–4, the study drugs were administered at the following doses: remifentanil 0.05–0.1 μg/kg/min and dexmedetomidine 0.2–0.7 μg/kg/h. The propofol infusion rate was adjusted to within the range of 25–75 μg/kg/min. If the patient or surgeon requested more sedation, 1–2 mg midazolam was additionally injected during the procedure. Before the surgical incision, all participants were administered 80 mg of 2% lidocaine as a local anesthetic on the skin. Depending on the surgical procedure, PI values, mean blood pressure (MBP), and heart rate (HR) were recorded at baseline, skin incision, dilator insertion, and skin suture. Intraoperative airway management was performed, including head tilting, chin lifting, jaw-thrust manipulation, and nasopharyngeal and laryngeal airway insertion. If airway management was required, the anesthetic dose was adjusted.

After finishing the surgery, all anesthetics including the study drugs were discontinued in the operating room. Then, the patient was transferred to the post anesthesia care unit (PACU). PI values, pain severity, OAA/S scale, and additional analgesic requirements were recorded. Pain severity was investigated using numerical rating scale (NRS) scores on a 10-point scale and patient satisfaction was recorded using 5-point Likert scale [8]. All variables began to measured approximately 5 min after study drugs were discontinued and were collected at 10-minute intervals from the admission to discharge from the PACU. Paracetamol 1 g (Kabi Paracetamol Injection, acetaminophen 10 mg/mL, Fresenius Kabi, Bad Homburg v.d.H. Germany) was additionally administered when the patient expressed surgical site pain with an NRS score ≥ 4 and the patient requested. Researcher who recorded the study variables in the PACU was blinded to the assigned group until all recordings were completed.

### Outcomes

The primary outcome was the comparison of PI values 30 min after admission to the PACU. To evaluate the lasting effect of the anesthetic used during sedation, PI measurement time of the primary outcome was set to 30 min after entering the PACU. The secondary outcomes were the comparison of NRS scores and PI values according to the time intervals in the PACU and the correlation of the PI and NRS scores in the PACU. In addition, the incidence of airway management during surgery and additional analgesic requirements in the PACU were investigated.

### Statistical analysis

The sample size was calculated based on unpublished clinical data. The mean (standard deviation) of PI value at 30 min after entering PACU was 2.81 (1.88) in dexmedetomidine group and 1.5 (1.38) in remifentanil group. To evaluate this at a significance level of 5% and power of 90%, we needed 35 patients in each group. Considering an expected dropout rate of 10%, 78 participants were required.

Continuous variables were expressed as mean (standard deviation) or median (interquartile range), as appropriate, and normality was assessed using the Shapiro-Wilk test. Categorical variables were presented as numbers (percentages). Intraoperative and postoperative continuous variables were compared using the t-test or Mann-Whitney U test as appropriate. Bonferroni corrections were applied for post-hoc analyses. Categorical variables were analyzed using Pearson’s chi-square test or Fisher’s exact test, as appropriate. Changes in PI in the PACU were analyzed using the Wilcoxon signed-rank test. The correlation between NRS scores and PI values in the PACU was analyzed using Pearson’s correlation coefficient. Statistical analysis was performed using SPSS 25.0 (SPSS Inc., Chicago, IL, USA). *P* < 0.05 was considered as statistically significant.

## Results

**Fig. 1:**
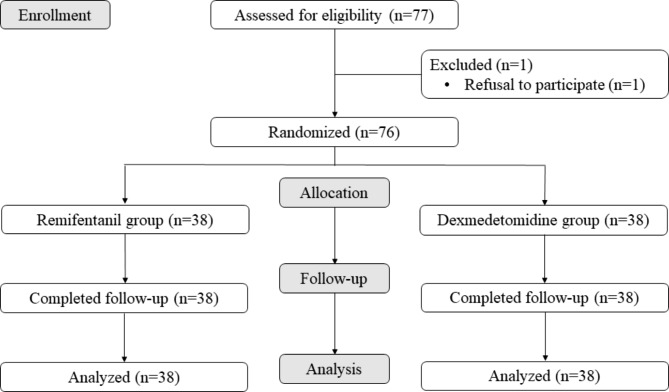
CONSORT Flow Diagram

Seventy-seven patients were enrolled, and one was excluded from the analysis due to refusal to participate in this study. Ultimately, 76 patients completed the trial (Fig. 1). Despite random assignment, there were significant differences in body weight and BMI between the two groups. Table 1 showed patient’s characteristics.

**Table 1:** Patient characteristics and intraoperative data

	Remifentanil (n = 38)	Dexmedetomidine (n = 38)	*P* value
Age, year	55.1 (10.0)	50.9 (10.1)	0.076
Gender, male	17 (44.7%)	16 (42.1%)	0.817
Weight, kg	57.3 (7.9)	61.8 (11.0)	0.041
Height, Cm	165.6 (8.0)	163.3 (10.0)	0.273
Body mass index, kg/m2	21.0 (3.3)	23.0 (2.3)	0.003
ASA PS classification, I/II	21/17	15/23	0.168
Diagnosis			0.073
Gastric ca.	2 (5.3%)	1 (2.6%)	
Breast ca.	9 (23.7%)	4 (10.5%)	
Colon ca.	8 (21.1%)	4 (10.5%)	
Ovary, cervix ca.	3 (7.9%)	1 (2.6%)	
Pancreas ca.	12 (31.6%)	6 (15.8%)	
Thyroid ca.	0	2 (5.3%)	
Rectal ca.	4 (10.5%)	18 (47.4%)	
Jejunal, intrahepatic bile duct ca.	0	2 (5.3%)	
Duration			
Anesthesia duration, min	59.7 (10.4)	52.8 (8.1)	0.002
Surgery duration, min	42.6 (13.2)	35.0 (8.0)	0.004
PACU staying, min	34.3 (1.9)	33.9 (2.0)	0.411
Drug consumption			
Propofol, mg	252.6 (67.7)	174.9 (44.0)	0.010
Study drug, mg	0.2 (0.1)	33.0 (23.5)	-
Midazolam, mg	1.6 (0.6)	1.6 (0.8)	0.942
Additional analgesics	0	0	-

Data expressed as mean (SD) or n (%)

ASA PS, American Society of Anesthesiologists physical status

Intraoperative MBP showed a significant difference between the two groups at each procedure [remifentanil group vs. dexmedetomidine group, baseline: 83.0 (72.0–90.0) mmHg vs. 85.0 (75.5–94.0) mmHg, *P* > 0.99; skin incision: 64.0 (57.0–68.0) mmHg vs. 75.0 (68.0–84.0) mmHg, *P* < 0.001; dilator insertion: 64.0 (60.0–69.0) mmHg vs. 76.0 (67.0–85.5) mmHg, *P* = 0.005; skin suture: 66.0 (60.0–70.0) mmHg vs. 77.0 (66.5–83.5) mmHg, *P* = 0.002; respectively]. There were no differences in HR between the two groups during intraoperative procedures [baseline: 72.0 (64.8–91.0) vs. 74.0 (64.5–85.0), *P* > 0.99; skin incision: 66.0 (85.3–79.3) vs. 64.0 (59.3–70.5), *P* > 0.99; dilator insertion: 68.0 (57.8–78.0) vs., 64.5 (59.5–69.3), *P* = 0.377; skin suture: 67.0 (59.0–76.0) vs. 62.0 (56.8–68.5), *P* = 0.288; respectively].

During intraoperative procedure, the PI values were not significantly different between the two groups [baseline: 1.6 (1.1–2.3) vs. 2.7 ( 1.3–4.5), *P* = 0.42; skin incision: 3.9 (2.6–5.9) vs. 3.5 (2.3–5.4), *P* > 0.99; dilator insertion: 3.4 (1.3–4.6) vs. 3.3 (2.1–5.3), *P* > 0.99; skin suture: 3.6 (1.7–4.8) vs. 2.8 (1.7–4.8), *P* > 0.99; respectively].

During staying at PACU, MBP showed statistical differences between the two study groups [entering the PACU: 107.2 (100.9–115.7) mmHg vs. 97.3 (85.8–110.1) mmHg, *P* = 0.063; 10 min: 105.3 (94.6–111.1) mmHg vs. 94.5 (86.1–107.1) mmHg, *P* = 0.046; 20 min: 109.3 (101.0–118.4) mmHg vs. 99.4 (87.7–111.7) mmHg, *P* = 0.040; 30 min: 107.2 (98.9–117.4) mmHg vs. 97.9 (86.4–107.1) mmHg, *P* = 0.011]. There were no statistical differences in HR [entering the PACU: 68.5 (62.5–77.0) vs. 63.0 (59.0–69.0), *P* = 0.096; 10 min: 67.0 (61.0–80.0) vs. 62.0 (59.0–67.3), *P* = 0.080; 20 min: 66.5 (59.8–80.0) vs. 62.0 (57.0–68.0), *P* = 0.106; 30 min: 67.0 (61.8–84.0) vs. 64.5 (56.8–71.0), *P* = 0.186].

**Fig. 2:**
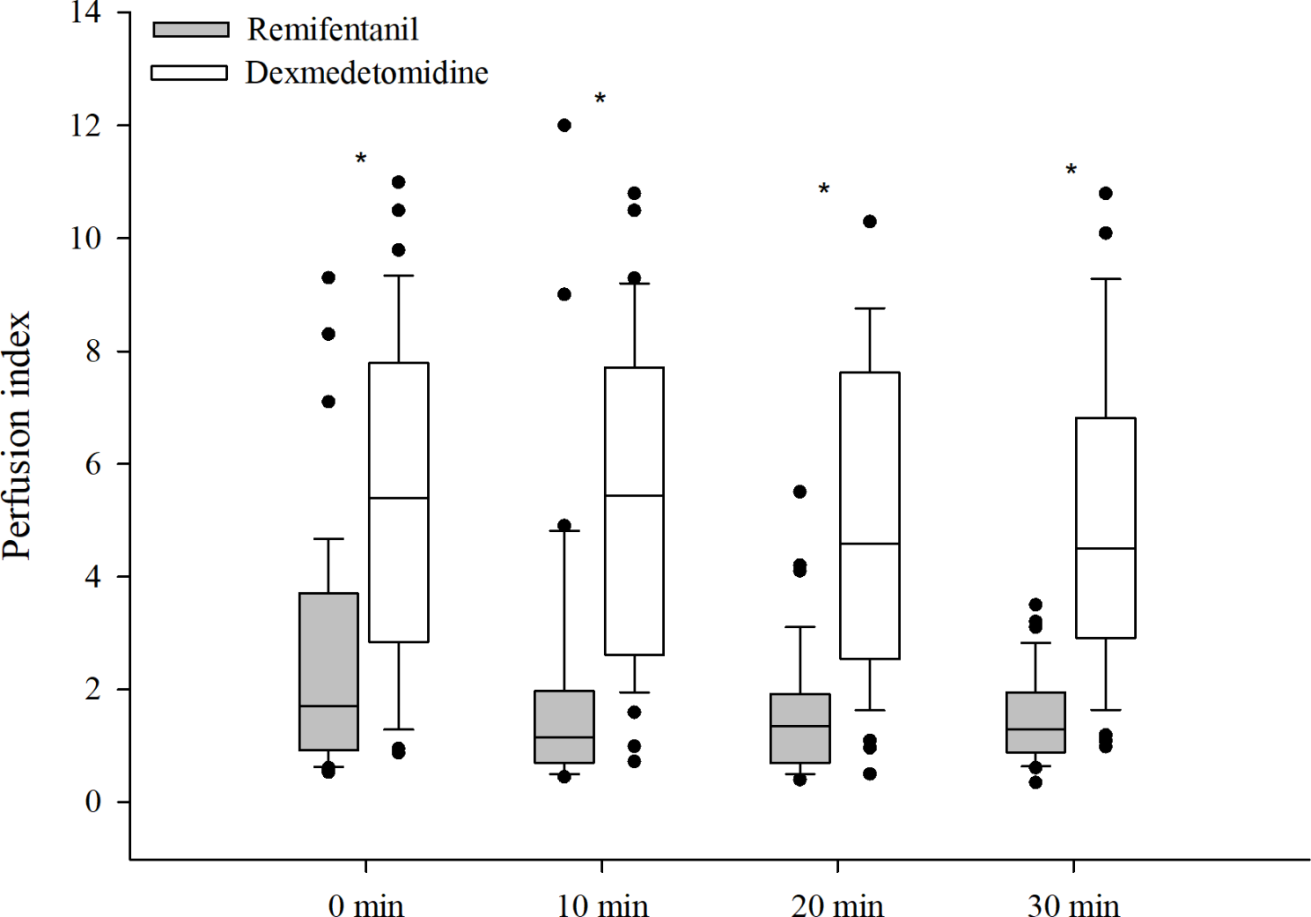
**PI values in post anesthesia care unit** Boxes represent the medians with the 25th/75th percentiles *, *P* < 0.05

The PI values at 30 min after admission to the PACU were significantly higher in the dexmedetomidine group than in the remifentanil group [remifentanil group:1.3 (0.9–2.0), dexmedetomidine group:4.5 (2.9–6.8) (median difference, 3; 95% CI, 2.1 to 4.2; *P* < 0.001] (Fig. 2). PI showed a significant difference between the two groups throughout the PACU stay (*P* < 0.001, all). The change in PI between admission and discharge of the PACU showed a statistically significant decrease in the remifentanil group [1.7 (0.9, 3.7) vs. 1.6 (1.0, 2.1), *P* = 0.022]. However, the change in PI was not significant in the dexmedetomidine group [5.4 (2.8, 7.8) vs. 4.5 (2.5, 6.8), *P* = 0.409].

The NRS scores measured at 10-minute intervals in the PACU showed no statistical differences between the two groups except for 30 min in the PACU (0 min, *P* > 0.99; 10 min, *P* > 0.99; 20 min, *P* = 0.064; 30 min, *P* = 0.004) (Table 2). There was a weak positive correlation between NRS score and PI in the PACU (correlation coefficient, 0.188; *P* = 0.01).

**Table 2:** NRS score and Perfusion index in PACU

	Remifentanil (n = 38)	Dexmedetomidine (n = 38)	*P* value
NRS score			
0 min	0 (0, 1.0)	0 (0, 1.0)	> 0.99
10 min	1.0 (0, 1.0)	0 (0, 2.0)	> 0.99
20 min	1.5 (0, 2.0)	0 (0, 2.0)	0.065
30 min	2.0 (0, 3.0)	0 (0, 2.0)	0.004
PI			
0 min	1.7 (0.9, 3.7)	5.4 (2.9, 7.8)	< 0.001
10 min	1.2 (0.5, 2.0)	5.8 (2.7, 7.8)	< 0.001
20 min	1.4 (0.7, 1.9)	4.9 (3.0, 7.3)	< 0.001
30 min	1.3 (0.9, 2.0)	4.5 (2.5, 6.8)	< 0.001

Data expressed as median (IQR).

*P* values were adjusted for multiple comparisons

NRS, numerical rating scale; PI, perfusion index; PACU, post anesthesia care unit

Except for the time immediately after entering the PACU, the OAA/S scale was not significantly different between the two study groups [remifentanil group, 0 min: 4 (4, 4), 10 min: 5 (5, 5), 20 min: 5 (5, 5), 30 min: 5 (5, 5); dexmedetomidine group, 0 min: 4 (2.8, 4), 10 min: 5 (4, 5), 20 min: 5 (5, 5), 30 min: 5 (5, 5); 0 min: *P* = 0.020, 10 min: *P* = 0.063, 20 min: *P* > 0.99, 30 min: *P* > 0.99, respectively].

The incidence of airway management was not statistically different during surgery [remifentanil, 4/38 (10.5%); dexmedetomidine, 2/38 (5.3%); *P* = 0.674]. Only one patient in the remifentanil group (2.6%) required rescue analgesics in the PACU.

## Discussion

In this study, monitored anesthesia care using dexmedetomidine and propofol showed significantly lower NRS score 30 min after administration in the PACU compared to remifentanil and propofol. The PI values 30 min after admission to the PACU were significantly higher when dexmedetomidine and propofol were used. The PI and NRS scores showed weak positive correlation during staying at PACU.

Numerous studies, investigated the role of dexmedetomidine as an anesthetic adjuvant, suggested that dexmedetomidine has many advantages compared to propofol or remifentanil, such as better hemodynamic stability, less respiratory depression, and long-lasting postoperative analgesic effects [3, 9, 10]. In line with this, our results showed lower NRS scores in dexmedetomidine group than remifentanil group at 30 min after administration in the PACU. There are several possible reasons for not showing a difference in NRS scores at other measurement time points. The postoperative pain of our participants may not have been severe. Since our goal was to find an effective adjuvant for monitoring anesthesia care in ambulatory surgery and use it in clinical practice, there were limitations in selecting the target group. In addition, we were concerned that the patient’s description of postoperative pain might be unclear due to the residual sedation effect of the study drugs. Considering this, the OAA/S scale was also continuously recorded in the PACU. Consequently, it was found that the level of consciousness of the two groups was similar except for the time of entering the PACU. Therefore, we suggest that the residual sedative effect of dexmedetomidine did not cause the patients to express their pain indefinitely. PI after sedation may not accurately reflect the patient’s pain severity and analgesia until complete recovery of level of consciousness.

It has been reported that PI can be used as an indicator in various situations during the perioperative period, including the success of regional anesthesia, hemodynamic stability, and recovery after anesthesia [11–14]. In addition, Chu et al., who tried to apply PI as an indicator of recovery in PACU, reported that an increase of PI value after admission of the PACU is useful as a criterion for pain assessment and discharge from the PACU [15]. In our study, PI values between admission and discharge in the PACU remained high when dexmedetomidine was used, whereas a significant decrease was seen in the remifentanil group. Furthermore, PI and NRS scores only showed a weak positive correlation. The context-sensitive half time was 3.2 min for remifentanil regardless of infusion time, [16] 4 min after a 10-minute infusion to 250 min after an 8 h infusion of dexmedetomidine [17]. Considering the duration of action of dexmedetomidine and remifentanil, it was expected a difference of the duration of analgesic effect of the two drugs. In addition, we suggest that sympatholytic action of dexmedetomidine may have contributed to the increased PI value. Remifentanil exhibits analgesic effects through vasodilation through Ca + channel inhibition [18]. Whereas, dexmedetomidine induces vasodilation through stimulation of α2-adrenoceptor in endothelial cells [19] and reduction of sympathetic nervous system tone [20]. Although it has not been clearly elucidated which are more potent, it is considered that the difference in PI between dexmedetomidine and remifentanil was caused by a combination of differences in vasodilation associated with sympatholytic action and analgesia. Unfortunately, our study did not reveal a clear mechanism for this. Thus, it may be difficult to simply use a PI value as an indicator of pain. Clinicians should be cautious in interpreting the PI when using sympatholyic agents such as dexmedetomidine.

Despite several deficiencies in our study, dexmedetomidine has many advantages as an anesthetic adjuvant during sedation. It causes less respiratory depression than other anesthetic adjuvants. Park et al. reported that the combination of dexmedetomidine and remifentanil showed hemodynamic stability and had less respiratory depression than the propofol/remifentanil combination during hysteroscopy performed under monitored anesthesia care [21]. Although there was no statistically significant difference in the current study, the incidence of rescue airway management was higher with remifentanil than with dexmedetomidine (10.5% vs. 5.3%). In addition, dexmedetomidine has an antisialagogue effect that may prevent airway irritation or aspiration caused by secretion during sedation [22].

This study had several limitations. First, we conducted this study only for chemoport insertion. Chemoport insertion is not painful enough, and some centers perform it only under local anesthesia. However, we have the advantage of excluding possible biases from anesthesia combinations, such as neuraxial anesthesia and sedation, owing to the nature of using only sedative anesthetics during surgery. Second, there were statistical differences in body weight and BMI in our participants. However, body weight and/or BMI had little to do with PI [23]. Thus, their effect on PI would have been minor. Third, this study compared the effects of these two drugs in combination with propofol. Thus, these results may have been derived from the interactions with propofol. Both dexmedetomidine and remifentanil are independent sedative and analgesic agents, respectively. The use of dexmedetomidine or remifentanil alone may have led to different results. In particular, dexmedetomidine is widely used for procedural sedation even when used alone. Further studies on postoperative pain control in patients undergoing ambulatory surgery are required. Lastly, the expression of the pain severity in PACU might be ambiguous due to the latent sedative effect of the study drugs associated with different duration. However, as shown in our results, there was no difference in the level of consciousness, represented by OAA/S scale, in PACU.

In summary, although PI values in the PACU were higher when dexmedetomidine and propofol were used, PI values may be to be used as a sole indicator of pain.

## Data Availability

The data associated with the paper are not publicly available but are available from the corresponding author on reasonable request.

## References

[1] Komatsu R, Turan AM, Orhan-Sungur M, McGuire J, Radke OC, Apfel CC (2007). Remifentanil for general anaesthesia: a systematic review. Anaesthesia.

[2] Kaur M, Singh PM (2011). Current role of dexmedetomidine in clinical anesthesia and intensive care. Anesth Essays Res.

[3] Kim D, Jeong JS, Park H, Sung KS, Choi SJ, Gwak MS, Kim GS, Hahm TS, Ko JS (2019). Postoperative pain control after the use of dexmedetomidine and propofol to sedate patients undergoing ankle surgery under spinal anesthesia: a randomized controlled trial. J Pain Res.

[4] Wu CL, Berenholtz SM, Pronovost PJ, Fleisher LA (2002). Systematic review and analysis of postdischarge symptoms after outpatient surgery. Anesthesiology.

[5] Coutrot M, Dudoignon E, Joachim J, Gayat E, Vallée F, Dépret F (2021). Perfusion index: physical principles, physiological meanings and clinical implications in anaesthesia and critical care. Anaesth Crit Care Pain Med.

[6] Chu C-L, Huang Y-Y, Chen Y-H, Lai L-P, Yeh H-M (2018). An observational study: the utility of perfusion index as a discharge criterion for pain assessment in the postanesthesia care unit. PLoS ONE.

[7] Mohamed S, Mohamed N, Rashwan D (2015). Pulse co-oximetry perfusion index as a tool for acute postoperative pain assessment and its correlation to visual analogue pain score. Res Opin Anesth Intensive Care.

[8] Likert R (1932). A technique for the measurement of attitudes. Archives of Psychology.

[9] Chan AK, Cheung CW, Chong YK (2010). Alpha-2 agonists in acute pain management. Expert Opin Pharmacother.

[10] Gurbet A, Basagan-Mogol E, Turker G, Ugun F, Kaya FN, Ozcan B (2006). Intraoperative infusion of dexmedetomidine reduces perioperative analgesic requirements. Can J Anaesth.

[11] Kim D, Jeong JS, Park MJ, Ko JS (2020). The effect of epinephrine on the perfusion index during ultrasound-guided supraclavicular brachial plexus block: a randomized controlled trial. Sci Rep.

[12] Toyama S, Kakumoto M, Morioka M, Matsuoka K, Omatsu H, Tagaito Y, Numai T, Shimoyama M (2013). Perfusion index derived from a pulse oximeter can predict the incidence of hypotension during spinal anaesthesia for caesarean delivery. Br J Anaesth.

[13] Krishnamohan A, Siriwardana V, Skowno JJ (2016). Using a pulse oximeter to determine clinical depth of anesthesia-investigation of the utility of the perfusion index. Paediatr Anaesth.

[14] Højlund J, Agerskov M, Clemmesen CG, Hvolris LE, Foss NB (2020). The Peripheral Perfusion Index tracks systemic haemodynamics during general anaesthesia. J Clin Monit Comput.

[15] Chu C-L, Huang Y-Y, Chen Y-H, Lai L-P, Yeh H-M (2018). An observational study: the utility of perfusion index as a discharge criterion for pain assessment in the postanesthesia care unit. PLoS ONE.

[16] Beers R, Camporesi E (2004). Remifentanil update: clinical science and utility. CNS Drugs.

[17] Iirola T, Ihmsen H, Laitio R, Kentala E, Aantaa R, Kurvinen JP, Scheinin M, Schwilden H, Schüttler J, Olkkola KT (2012). Population pharmacokinetics of dexmedetomidine during long-term sedation in intensive care patients. Br J Anaesth.

[18] Hu ZY, Lin PT, Liu J, Liao DQ (2008). Remifentanil induces L-type Ca2 + channel inhibition in human mesenteric arterial smooth muscle cells. Can J Anaesth.

[19] Talke P, Lobo E, Brown R (2003). Systemically administered alpha2-agonist-induced peripheral vasoconstriction in humans. Anesthesiology.

[20] Noseir RK, Ficke DJ, Kundu A, Arain SR, Ebert TJ (2003). Sympathetic and vascular consequences from remifentanil in humans. Anesth Analg.

[21] Park S, Choi SL, Nahm FS, Ryu JH, Do SH (2020). Dexmedetomidine-remifentanil vs propofol-remifentanil for monitored anesthesia care during hysteroscopy: Randomized, single-blind, controlled trial. Med (Baltim).

[22] Khan ZP, Ferguson CN, Jones RM (1999). alpha-2 and imidazoline receptor agonists. Their pharmacology and therapeutic role. Anaesthesia.

[23] Boly CA, Venhuizen M, Dekker NAM, Vonk ABA, Boer C, van den Brom CE. Comparison of Microcirculatory Perfusion in Obese and Non-Obese Patients Undergoing Cardiac Surgery with Cardiopulmonary Bypass. J Clin Med. 2021, 10(3). 10.3390/jcm10030469 PMC7865338 33530543

